# *Croton gratissimus* leaf extracts inhibit cancer cell growth by inducing caspase 3/7 activation with additional anti-inflammatory and antioxidant activities

**DOI:** 10.1186/s12906-018-2372-9

**Published:** 2018-11-14

**Authors:** Emmanuel Mfotie Njoya, Jacobus N. Eloff, Lyndy J. McGaw

**Affiliations:** 1 Phytomedicine Programme, Department of Paraclinical Sciences, Faculty of Veterinary Science, University of Pretoria, Private Bag X04, Onderstepoort, Pretoria, 0110 South Africa; 2 Department of Biochemistry, Faculty of Science, University of Yaoundé I, P.O. Box 812, Yaoundé, Cameroon

**Keywords:** *Croton gratissimus*, Free radicals, Nitric oxide, 15-lipoxygenase, Cytotoxicity, Caspases

## Abstract

**Background:**

*Croton* species (Euphorbiaceae) are distributed in different parts of the world, and are used in traditional medicine to treat various ailments including cancer, inflammation, parasitic infections and oxidative stress related diseases. The present study aimed to evaluate the antioxidant, anti-inflammatory and cytotoxic properties of different extracts from three *Croton* species.

**Methods:**

Acetone, ethanol and water leaf extracts from *C. gratissimus*, *C. pseudopulchellus,* and *C. sylvaticus* were tested for their free radical scavenging activity. Anti-inflammatory activity was determined via the nitric oxide (NO) inhibitory assay on lipopolysaccharide (LPS)-stimulated RAW 264.7 macrophages, and the 15-lipoxygenase inhibitory assay using the ferrous oxidation-xylenol orange assay. The cytotoxicity of the extracts was determined on four cancerous cell lines (A549, Caco-2, HeLa, MCF-7), and a non-cancerous African green monkey (Vero) kidney cells using the tetrazolium-based colorimetric (MTT) assay. The potential mechanism of action of the active extracts was explored by quantifying the caspase-3/− 7 activity with the Caspase-Glo® 3/7 assay kit (Promega).

**Results:**

The acetone and ethanol leaf extracts of *C. pseudopulchellus* and *C. sylvaticus* were highly cytotoxic to the non-cancerous cells with LC_50_ varying between 7.86 and 48.19 μg/mL. In contrast, the acetone and ethanol extracts of *C. gratissimus* were less cytotoxic to non-cancerous cells and more selective with LC_50_ varying between 152.30 and 462.88 μg/mL, and selectivity index (SI) ranging between 1.56 and 11.64. Regarding the anti-inflammatory activity, the acetone leaf extract of *C. pseudopulchellus* had the highest NO inhibitory potency with an IC_50_ of 34.64 μg/mL, while the ethanol leaf extract of the same plant was very active against 15-lipoxygenase with an IC_50_ of 0.57 μg/mL. A linear correlation (r<0.5) was found between phytochemical contents, antioxidant, anti-inflammatory and cytotoxic activities of active extracts. These extracts induced differentially the activation of caspases − 3 and − 7 enzymes in all the four cancerous cells with the highest induction (1.83-fold change) obtained on HeLa cells with the acetone leaf extract of *C. gratissimus*.

**Conclusion:**

Based on their selective toxicity, good antioxidant and anti-inflammatory activities, the acetone and ethanol leaf extracts of *C. gratissimus* represent promising alternative sources of compounds against cancer and other oxidative stress related diseases.

**Electronic supplementary material:**

The online version of this article (10.1186/s12906-018-2372-9) contains supplementary material, which is available to authorized users.

## Background

Oxidative stress results from an imbalance between the production of free radicals and the ability of the body to counteract or detoxify their harmful effects through neutralization by antioxidants [1]. The free radical theory of aging developed by Denham Harman is based on the concept that damage accumulates throughout the entire lifespan and causes age dependent disorders including diabetes, atherosclerosis, neurodegenerative diseases and cancer [2, 3]. Cancer development is characterized by redox imbalance with a shift towards oxidative conditions. In fact, free radicals can bind through electron pairing with macromolecules such as proteins, phospholipids and DNA in normal cells to cause protein and DNA damage along with lipid peroxidation [1]. Consequently, the accumulation of these cellular disorders may cause mutation and lead to various disturbances in the cell metabolism, which can result in deregulated cell growth, and finally carcinoma [4]. Antioxidants are helpful in reducing and preventing damage caused by free radicals because of their ability to donate electrons, which neutralize the radicals without forming another. This property has led to the hypothesis that antioxidants, with their ability to decrease the level of free radicals, might lessen the radical damage causing chronic diseases, and even radical damage responsible for aging and cancer. Antioxidant phytochemicals found in vegetables, fruits and medicinal plants have been reported to be responsible for health benefits such as the prevention and treatment of chronic diseases caused by oxidative stress [5]. Many antioxidant phytochemicals have been associated with anti-cancer activities, and this includes curcumin from turmeric, genistein from soybean, tea polyphenols from green tea, resveratrol from grapes, sulforaphane from broccoli, isothiocyanates from cruciferous vegetables, silymarin from milk thistle, diallyl sulfide from garlic, lycopene from tomato, rosmarinic acid from rosemary, apigenin from parsley, and gingerol from gingers [6].

During the last two decades, it has been revealed that oxidative stress can lead to chronic inflammation, which in turn could mediate most chronic diseases including cancer. Chronic inflammation is usually associated with an increased risk of several human cancers [7]. Indeed, the relationship between inflammation and cancer has been suggested by epidemiological and experimental data, and confirmed by the fact that anti-inflammatory therapies were also efficient in cancer prevention and treatment [8, 9].

The genus *Croton* belongs to the family Euphorbiaceae, and is a diverse and complex group of plants ranging from herbs and shrubs to trees. *Croton* species can be found in different parts of the world, and some of the most popular uses include treatment of cancer, constipation, diabetes, digestive problems, dysentery, external wounds, intestinal worms, pain, ulcers and weight loss [10]. *Croton sylvaticus* Hochst. is a fast-growing and decorative tree, which is widely used in the management of inflammatory conditions, infections and oxidative stress related diseases. In Tanzania and Kenya, the decoction of the leaves and root bark of *C. sylvaticus* is used in traditional medicine against tuberculosis (TB), inflammation, as a purgative, as a wash for body swelling caused by kwashiorkor or by tuberculosis, and for the treatment of malaria [11]. Previous reports showed the acetylcholinesterase inhibitory activity of the ethyl acetate leaf extract of *C. sylvaticus* and isolated compounds [12]. Other compounds isolated from this plant have antiplasmodial activity [13], and low to high toxicity observed in the brine shrimp larval lethality test [11]. *Croton gratissimus* Burch. (synonym *C. zambesicus* Müll.Arg.) is native to tropical west and central Africa, and is used to treat fever, dysentery and convulsions [14]. The leaf decoction is used in Benin as anti-hypertensive, anti-microbial (against urinary infections) and to treat malaria-linked fever [15]. Some compounds, named cembranolides isolated from leaf extracts of *Croton gratissimus*, have moderate activity against ovarian cancer cell lines and *Plasmodium falciparum* [16, 17]. *Croton pseudopulchellus* Pax, originating from southern Africa, is widely distributed in tropical East and West Africa. This *Croton* species is used in southern and central parts of South Africa against TB symptoms such as coughs, fever and blood in sputum [18]. Based on their diverse uses in traditional medicine against various diseases in which excess production of free radicals or inflammation is implicated, the present study aims to evaluate the antioxidant, anti-inflammatory and cytotoxic properties of three *Croton* species extracted using different solvents.

## Materials and methods

### Plant material and extraction

Fresh leaves of the three *Croton* species were collected at the Lowveld Botanical Gardens, Nelspruit, Mpumalanga (South Africa) in January 2016. The plant materials were dried at room temperature in a well-ventilated room for two weeks. The dried materials were ground to fine powder and stored in honey jars in the dark until use. Herbarium specimens for each of the plant species were prepared, and identification was made by Mrs. Elsa van Wyk and Ms. Magda Nel of the HGWJ Schweickerdt Herbarium (PRU), University of Pretoria. The identification numbers of plant species are presented in Table 1. Powder (100 g) from each plant was extracted by maceration in 1000 mL of different solvents (water, acetone and ethanol). The mixtures were covered and left overnight at room temperature. Each mixture was filtered through Whatman No.1 filter paper into pre-weighed honey jars and the filtrates obtained from acetone and ethanol extraction were concentrated under reduced pressure using a rotary evaporator at 40 °C to obtain a residue which constituted the crude extract. The water filtrate was dried in a ventilated oven at 50–55 °C until complete evaporation of water. The extraction process was repeated three times with fresh solvent. The honey jars containing the crude extracts were weighed again to determine the percentage yield of the crude extracts (Table 1). The dried extracts were stored in a cold room (4 °C) until use.

**Table 1:** Herbarium specimen identification and yield of crude extracts from the three *Croton* species

Plant name	Family name	Herbarium specimen no.	Yield of extraction (%)		
			Water	Acetone	Ethanol
*Croton gratissimus* Burch.	Euphorbiaceae	PRU/122516	3.29	5.15	6.23
*Croton pseudopulchellus* Pax	Euphorbiaceae	PRU/122519	4.65	7.63	8.95
*Croton sylvaticus* Hochst.	Euphorbiaceae	PRU/122523	4.11	6.18	7.59

### Phytochemical analysis

#### Total phenolic content

The total phenolic content (TPC) of different extracts was determined using the Folin-Ciocalteu method adapted to a 96-well microplate as described by Zhang et al. [19]. The reaction mixture was prepared by adding respectively 20 μL of each extract (5 mg/mL in DMSO), 100 μL of Folin-Ciocalteu reagent (1 mL of Folin-Ciocalteu reagent in 9 mL of distilled water), and 80 μL 7.5% Na_2_CO_3_ solution in deionized water. The mixture was then incubated in the dark at room temperature (25 °C) for 30 min, and the absorbance was read at 765 nm on a microplate reader (Epoch, BioTek). The total phenolic content was estimated from a gallic acid (GA) calibration curve (10–100 mg/L; *y = 0.6886x + 0.0884; R*^*2*^ *= 0.9901*), and results were expressed as milligram of gallic acid equivalent (GAE) per gram of extract.

#### Total flavonoid content

The total flavonoid content (TFC) of different extracts was determined using the aluminium chloride spectrophotometric method based on the formation of aluminium-flavonoid complexes [20]. The reaction mixture was prepared by mixing 2 mL of each extract (0.3 mg in 1 mL of methanol), 0.1 mL of aluminium chloride hexahydrate solution (10% aqueous AlCl_3_ solution), 0.1 mL of 1 M potassium acetate and 2.8 mL of deionized water. The mixture was shaken and incubated at room temperature (25 °C) for 10 min, and 200 μL of each mixture was transferred to 96-well microplate. The absorbance was measured at 415 nm using a microplate reader (Epoch, BioTek). A calibration curve was plotted from the absorbance of quercetin (0.005–0.1 mg/mL; *y = 9.0545x – 0.0142; R*^*2*^ *= 0.9999*), and the total flavonoid content was expressed as milligram of quercetin equivalent (QE) per gram of extract.

### Antioxidant assays

#### The 2,2-diphenyl-1-picrylhydrazyl (DPPH) assay

The technique described by Brand-Williams et al. [21] with some modifications was applied for the determination of the DPPH scavenging capacity of extracts. Briefly, the extracts (40 μL) were serially diluted with methanol on a 96-well plate, followed by the addition of the DPPH solution (160 μL) prepared at 25 μg/mL. The mixture was incubated at room temperature in the dark for 30 min and the absorbance was measured at 517 nm using a microplate reader (Epoch, BioTek). Ascorbic acid and trolox were used as positive controls, methanol plus DPPH as negative control, and sample without DPPH as blank. The DPPH scavenging capacity was calculated at each concentration according to the formula (1) below:

1 $$ \mathrm{Scavenging}\ \mathrm{capacity}\ \left(\%\right)=\frac{\mathrm{Absorbance}\ \left(\mathrm{control}\right)\hbox{-} \mathrm{Absorbance}\ \left(\mathrm{sample}\right)}{\mathrm{Absorbance}\ \left(\mathrm{control}\right)}\times 100 $$

The inhibitory concentration (IC_50_) was determined by plotting a non-linear curve of percentage DPPH scavenging capacity against the logarithm of different concentrations of the extract.

#### The 2,2′-azino-bis (3-ethylbenzothiazoline-6-sulfonic acid) (ABTS) assay

The method described by Re et al. [22] with some modifications was used for the determination of the ABTS radical scavenging capacity of the extracts. Firstly, the reaction solution was prepared by mixing a solution of ABTS (7 mM) with a solution of potassium persulfate (2.45 mM) at room temperature for 12 to 16 h. The optical density of the reaction solution containing the ABTS radical produced was calibrated to 0.70 ± 0.02 at 734 nm before use. Secondly, the extracts (40 μL) were serially diluted with methanol, followed by the addition of the ABTS radical (160 μL), and the optical density was measured after 5 min at 734 nm using a microplate reader (Epoch, BioTek). Two positive controls (trolox and ascorbic acid) were used. Methanol plus ABTS radical was used as negative control while extract without ABTS was considered as the blank. The percentage of ABTS scavenging capacity was calculated at each concentration according to the formula (1) above, and the inhibitory concentrations (IC_50_) values were determined as indicated in the previous paragraph.

### Anti-inflammatory assays

#### Nitric oxide inhibitory assay

The method published by Dzoyem and Eloff [23] was used to determine the nitric oxide inhibitory activity of the extracts. The RAW 264.7 macrophages were obtained from the American Type Culture Collection (ATCC) (Rockville, MD, USA), and were grown at 37 °C with 5% CO_2_ in a humidified environment in Dulbecco’s Modified Eagle’s Medium (DMEM) high glucose (4.5 g/L) containing L-glutamine (4 mM) and sodium pyruvate (Hyclone™) supplemented with 10% (*v*/v) fetal bovine serum (Capricorn Scientific Gmbh, South America) and 1% penicillin-streptomycin-fungizone (PSF). Nitric oxide (NO) production by RAW 264.7 macrophages was measured using the Griess reagent (Sigma Aldrich, Germany) after 24 h of lipopolysaccharide (LPS) stimulation in the presence or absence of the extracts or quercetin used as positive control. Briefly, the RAW 264.7 macrophages were inoculated at a density of 2 × 10^4^ cells per well in 96 well-microtitre plates, and the cells were left overnight to allow attachment to the bottom of the plate. The cells were treated with different concentrations of the extracts dissolved in DMSO with the final concentration of DMSO not exceeding 0.5%. Thereafter, the cells were stimulated by addition of LPS at a final concentration of 1 μg/mL per well. The cells treated with only LPS were considered as the negative control. After 24 h of incubation at 37 °C with 5% CO_2_ in a humidified environment, the supernatant (100 μL) from each well of the 96-well microtitre plates were transferred into new 96-well microtitre plates, and an equal volume of Griess reagent (Sigma Aldrich, Germany) was added. The mixture was left in the dark at room temperature for 15 min, and the absorbance was determined at 550 nm on a microplate reader (Synergy Multi-Mode Reader, BioTek). The quantity of nitrite was determined from a sodium nitrite standard curve. The percentage of NO inhibition was calculated based on the ability of each extract to inhibit nitric oxide production by RAW 264.7 macrophages compared with the control (cells treated with LPS without extract). In addition, the cell viability was determined using the 3-(4,5-dimethythiazol- 2-yl)-2,5-diphenyl tetrazolium bromide (MTT) assay [24]. The culture medium was aspirated from the plates, and replaced by fresh medium (200 μL) with 30 μL of thiazolyl blue tetrazolium bromide (5 mg/mL) dissolved in phosphate buffered saline. After incubation for 4 h, the medium was gently aspirated, and the formazan crystals were dissolved in 50 μL of DMSO and kept in the dark for 15 min at room temperature. The absorbance was measured spectrophotometrically at 570 nm on a microplate reader (Synergy Multi-Mode Reader, BioTek).

### Inhibition of soybean 15-lipoxygenase (15-LOX) enzyme

The assay was performed according to the procedure of Pinto et al. [25] with slight modifications to the microtitre plate format. The assay is based on the formation of the complex Fe^3+^/xylenol orange with absorption at 560 nm. The 15-lipoxygenase (15-LOX) enzyme from soybean (Sigma Aldrich, Germany) was incubated with different concentrations of extracts or quercetin used as standard inhibitor (both serially diluted from 0.78 to 100 μg/mL) at 25 °C for 5 min. The substrate, linoleic acid (final concentration, 140 μM) prepared in Tris-HCl buffer (50 mM, pH 7.4), was added and the mixture was incubated at 25 °C for 20 min in the dark. The assay was terminated by the addition of 100 μL of FOX reagent [sulfuric acid (30 mM), xylenol orange (100 μM), iron (II) sulfate (100 μM) in methanol/water (9:1)]. The negative control was made of the enzyme 15-LOX solution, buffer, substrate and FOX reagent while the blanks contained the enzyme 15-LOX and buffer, but the substrate was added after the FOX reagent. The lipoxygenase inhibitory activity was evaluated by calculating the percentage of the inhibition of hydroperoxide production from the changes in absorbance values at 560 nm after 30 min at 25 °C as indicated in the formula (2) below.

2 $$ \mathrm{Percentage}\ \mathrm{LO}\ \mathrm{X}\ \mathrm{inhibition}\ \left(\%\right)=\frac{\mathrm{Absorbance}\ \left(\mathrm{control}\right)\hbox{-} \mathrm{Absorbance}\ \left(\mathrm{sample}\right)}{\mathrm{Absorbance}\ \left(\mathrm{control}\right)}\times 100 $$

The IC_50_ values of extracts or quercetin, which represent the concentration leading to 50% inhibition were calculated using the non-linear regression curve of the percentage (15-LOX) inhibition against the logarithm of concentrations tested.

### Cytotoxicity assay

#### Cell culture

The four cancer cell lines (MCF-7: human breast adenocarcinoma cells; HeLa: human cervix adenocarcinoma cells; Caco-2: human epithelial colorectal adenocarcinoma cells; A549: human epithelial lung adenocarcinoma cells) were obtained from the American Type Culture Collection (ATCC) (Rockville, MD, USA). These cells were grown at 37 °C with 5% CO_2_ in a humidified environment in Dulbecco’s Modified Eagle’s Medium (DMEM) high glucose (4.5 g/L) containing L-glutamine (4 mM) and sodium pyruvate (Separations, RSA) supplemented with 10% (*v*/v) fetal bovine serum (Capricorn Scientific Gmbh, South America). Non-cancerous African green monkey (Vero) kidney cells (obtained from ATCC) were maintained at 37 °C and 5% CO_2_ in a humidified environment in Minimal Essential Medium (MEM) containing L-glutamine (Lonza, Belgium) supplemented with 5% fetal bovine serum (Capricorn Scientific Gmbh, South America) and 1% gentamicin (Virbac, RSA).

### Cell treatment and assay procedure

The cells were seeded at a density of 10^4^ cells per well on 96-well microtitre plates, and were left overnight to allow attachment. After this, the cells were treated with different concentrations of extracts dissolved in dimethyl sulfoxide (DMSO), and further diluted in fresh culture medium. In each experiment, the highest concentration of DMSO (negative control) in the medium was 0.5%. After incubation for 48 h at 37 °C with 5% CO_2_, the culture medium was discarded, and replaced by fresh medium (200 μL) with 30 μL of thiazolyl blue tetrazolium bromide (5 mg/mL) dissolved in phosphate buffered saline. The medium was gently aspirated after 4 h of incubation, and the formazan crystals were dissolved in 50 μL of DMSO, and kept in the dark for 15 min at room temperature. The absorbance was measured spectrophotometrically at 570 nm on a microplate reader (Synergy Multi-Mode Reader, BioTek). The viability of cells treated with the extracts was calculated for each concentration compared to the negative control. The 50% inhibitory concentrations (IC_50_) for cancer cell lines and the 50% lethal concentrations (LC_50_) for the non-cancerous cells were determined by plotting the non-linear regression curve of percentage of cell survival versus the logarithm of concentrations of each extract. The selectivity index (SI) values were calculated for each extract by dividing the LC_50_ of the non-cancerous cell against the IC_50_ of each cancer cell type in the same units.

### Evaluation of the induction of apoptosis on cancer cells

The induction of apoptosis by the most active extracts from each plant was evaluated by measuring the caspase 3/7 activity on different cancer cell lines with the Caspase-Glo® 3/7 assay kit (Promega). All four cancer cell lines were seeded at a density of 10^4^ cells per well on 96-well microtitre plates, and were allowed to adhere overnight. These cells were treated with the extracts at different concentrations (½ × IC_50_, IC_50_ and 2 × IC_50_) or DMSO (0.5%) as negative control, and the plates were incubated at 37 °C with 5% CO_2_ for 24 h. After treatment, the Caspase-Glo® 3/7 was prepared according to manufacturer’s guidelines, and 100 μL of the reagent was added per well and incubated for 1 h at room temperature in the dark. Following this incubation, the luminescence was measured on a microplate reader (Synergy Multi-Mode Reader, BioTek). The data was analysed, and expressed as percentage of the untreated cells (control) and fold change.

### Statistical analysis

All experiments were performed in triplicate, and the results are presented as mean ± standard error of mean (SEM) values. Statistical analysis was carried out with GraphPad Instat 3.0 software. The Student–Newman–Keuls test was used to determine *P*-values for the differences observed between the extracts while Dunnett’s test was used to compare the extracts with the control. Results were considered significantly different when *P*< 0.05.

## Results

### Yield of extraction and phytochemical content of crude extracts

The voucher specimen numbers (PRU) and the yield of extraction of each plant material in a particular solvent are summarized in Table 1. The highest yield of extraction was observed with *C. pseudopulchellus* with all the three solvents used. Extraction with ethanol had the highest yield of extraction among the plant species. The phytochemical content of all extracts is presented in Table 2, and significant differences have been noted between total phenolic content (TPC) and total flavonoid content (TFC) of the plant materials extracted with the three solvents used. Organic solvents (acetone and ethanol) extracted more of these compounds compared to water. The acetone leaf extract of *C. gratissimus* had the highest TPC with 222.29 mgGAE/g whereas the highest TFC was obtained with the acetone and ethanol leaf extracts of *C. sylvaticus* with 82.76 and 84.54 mgQE/g respectively.

**Table 2:** Phytochemical content, antioxidant activity, nitric oxide and 15-lipoxygenase inhibition of different extracts from *Croton* species and positive controls

Plant name	Extracts	Phytochemicals		IC_50_ (μg/mL)			
		TPC (mgGAE/g)	TFC (mgQE/g)	DPPH	ABTS	NO	15-LOX
*Croton gratissimus*	CGA	222.29 ± 3.90^a^	43.35 ± 0.26^a^	217.64 ± 3.46^a^	170.51 ± 4.95^a^	49.24 ± 0.93^a^	10.97 ± 1.19^a^
	CGE	180.61 ± 1.74^b^	44.39 ± 0.27^a^	32.18 ± 2.11^b^	34.95 ± 0.81^b^	51.93 ± 0.11^a^	2.58 ± 0.02^b^
	CGW	121.92 ± 1.78^c^	29.50 ± 1.21^b^	> 500	> 500	88.90 ± 0.57^b^	> 100
*Croton pseudopulchellus*	CPA	124.05 ± 2.00^c^	35.88 ± 0.40^c^	220.34 ± 4.98^a^	176.94 ± 2.26^a^	34.64 ± 0.06^c^	2.64 ± 0.23^b^
	CPE	84.28 ± 1.52^d^	35.62 ± 0.36^c^	205.96 ± 3.66^a^	144.01 ± 2.28^c^	53.49 ± 0.47^a^	0.57 ± 0.17^c^
	CPW	35.10 ± 1.44^e^	15.97 ± 0.87^d^	> 500	> 500	> 100	> 100
*Croton sylvaticus*	CSA	112.34 ± 1.29^c^	82.76 ± 1.57^e^	285.64 ± 2.81^c^	165.84 ± 7.91^a^	68.28 ± 0.32^d^	11.64 ± 1.26^a^
	CSE	180.88 ± 1.93^b^	84.54 ± 1.85^e^	252.19 ± 2.11^c^	134.96 ± 7.83^c^	78.91 ± 2.19^d^	2.12 ± 0.37^b^
	CSW	99.27 ± 0.18^f^	26.06 ± 0.96^b^	> 500	> 500	> 100	> 100
Positive controls	Ascorbic acid	ND	ND	1.92 ± 0.08^d^	3.92 ± 0.24^d^	ND	ND
	Trolox	ND	ND	2.21 ± 0.24^d^	4.64 ± 0.46^d^	ND	ND
	Quercetin	ND	ND	ND	ND	5.82 ± 0.63^e^	24.60 ± 0.49^d^

Data are presented as means of triplicate measurements ± standard error, superscript letters a-f represent statistical difference between data obtained, and for each parameter within a column of the above table, data with different letters mean significantly different at p < 0.05 while data with same letters are statistically not different.; ND = Not Determined. IC_50_: concentration required to inhibit the activity by 50% compared to untreated controls. CGA, CGE and CGW represent respectively acetone, ethanol and water extracts of *Croton gratissimus*. CPA, CPE and CPW represent respectively acetone, ethanol and water extracts of *Croton pseudopulchellus.* CSA, CSE and CSW represent respectively acetone, ethanol and water extracts of *Croton sylvaticus*. TPC: total phenolic content (mg of gallic acid equivalent per gram of extract) and TFC: total flavonoid content (mg of quercetin equivalent per gram of extract). DPPH: 2,2-diphenyl-1-picrylhydrazyl radical, ABTS: 2,2′-azino-bis (3-ethylbenzothiazoline-6-sulfonic acid) radical, NO: nitric oxide, 15-LOX: 15-lipoxygenase

## Antioxidant activity of extracts

Two antioxidant assays which involved the measurement of colour disappearance caused by free radicals such as DPPH and ABTS were used. As expected, the free radical scavenging activity of the extracts was concentration-dependent (data not shown) and the IC_50_ values determined are presented in Table 2. The antioxidant activity varies within extracts from the same plant and between extracts from different plants. It should be noted that a lower IC_50_ value indicates a stronger antioxidant potency of the sample tested. Therefore, the ethanol leaf extracts from all the three plants have good antioxidant potency when compared with acetone and water extracts from the same plant. Among all the extracts from the three plants, the ethanol leaf extract of *C. gratissimus* had the highest antioxidant potency with IC_50_ values of 32.18 and 34.95 μg/mL respectively for the DPPH and ABTS radical scavenging activity. Ascorbic acid and trolox, known as potent antioxidant compounds, had the best antioxidant potency with IC_50_ values of 1.92 and 3.92 μg/mL (ascorbic acid); 2.21 and 4.64 μg/mL (trolox) respectively for the DPPH and ABTS radical scavenging activity (Table 2).

## Anti-inflammatory activity of extracts

The anti-inflammatory activity of leaf extracts was determined using the nitric oxide (NO) and 15-lipoxygenase (15-LOX) inhibitory assays.

### Nitric oxide inhibitory effect of extracts on LPS-stimulated RAW 264.7 macrophages

**Fig. 1:**
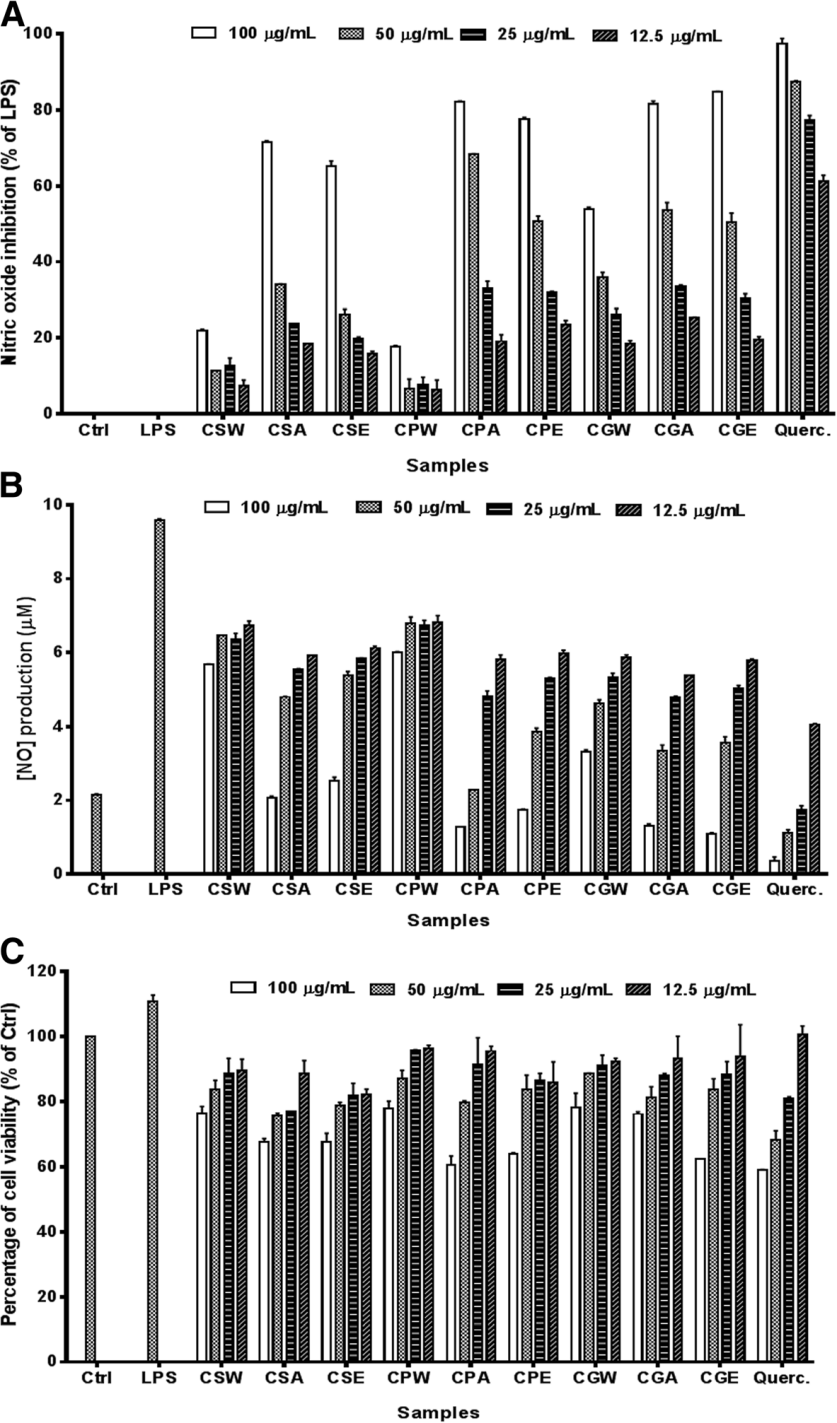
Activities of the extracts from three *Croton* species on the percentage of nitric oxide inhibition (**a**), nitric oxide production (**b**) and cell viability (**c**) on LPS-stimulated RAW 264.7 macrophages. Data are presented as means of triplicate measurements ± standard error. CSA, CSE and CSW represent respectively acetone, ethanol and water extracts of *Croton sylvaticus.* CPA, CPE and CPW represent respectively acetone, ethanol and water extracts of *Croton pseudopulchellus.* CGA, CGE and CGW represent respectively acetone, ethanol and water extracts of *Croton gratissimus*. Ctrl: control group (0.5% DMSO); LPS: lipopolysaccharide

All the extracts from the three *Croton* species had inhibitory activity on NO production in a concentration-dependent manner (Fig. 1a and b). Water leaf extracts of the three plants had the lowest NO inhibitory effect except for the water extract from *C. gratissimus* that had a good inhibitory activity. Acetone and ethanol leaf extracts of the plants had the highest NO inhibitory activity compared with their respective water leaf extracts. The IC_50_ values were calculated, and are presented in Table 2. Acetone leaf extracts from the three plants had the lowest IC_50_ values, which are not significantly different from the IC_50_ values obtained for the ethanol leaf extracts. However, the acetone leaf extract of *C. pseudopulchellus* had an IC_50_ value (34.64 μg/mL) significantly (*P* < 0.05) lower than the IC_50_ of the ethanol extract (53.49 μg/mL) from the same plant. The acetone leaf extract of *C. pseudopulchellus* therefore had the highest NO inhibitory potency. Quercetin, used as positive control, had the highest NO inhibitory potency with IC_50_ of 5.82 μg/mL.

The cell viability of LPS-stimulated RAW 264.7 macrophages after treatment with the extracts and quercetin is presented in Fig. 1c. The acetone and ethanol leaf extracts as well as quercetin were slightly cytotoxic on LPS-stimulated RAW 264.7 macrophages with percentage of cell viability varying between 62 and 96%. The water leaf extracts were less cytotoxic with cell viability greater than 76% at the highest concentration (100 μg/mL) tested.

### Lipoxygenase inhibitory activity of extracts

The ferrous oxidation-xylenol orange (FOX) assay was used to determine the 15-lipoxygenase inhibitory activity of different extracts from the three *Croton* species, and the IC_50_ values were determined using the non-linear regression curves (Additional file 1: Figure S1) and the results are presented in Table 2. All the extracts except the water extracts had better inhibitory activity against 15-lipoxygenase when compared to the positive control (quercetin). The IC_50_ values of the active extracts (acetone and ethanol) from the three plants varied between 0.57 and 11.64 μg/mL which is significantly (*P* < 0.05) different from quercetin (24.60 μg/mL). Ethanol leaf extracts were more active than acetone leaf extracts from the same plant species, thus suggesting that ethanol extracted more anti-lipoxygenase compounds than acetone. The highest lipoxygenase inhibitory activity was obtained with the ethanol leaf extract of *C. pseudopulchellus* (IC_50_ of 0.57 μg/mL).

### Selective cytotoxic effect of extracts on a non-cancerous cell versus cancerous cells

Different extracts were tested for cytotoxicity against four cancerous (A549, Caco-2, HeLa and MCF-7) cell types as well as the non-cancerous African green monkey (Vero) kidney cells, and the graphs of cell viability against the concentrations tested are presented in Additional file 2: Figure S2, Additional file 3: Figure S3, Additional file 4: Figure S4, Additional file 5: Figure S5 and Additional file 6: Figure S6 respectively. The LC_50_ and IC_50_ values of extracts were determined from concentration-dependent graphs, and are presented in Table 3. Water leaf extracts had the lowest cytotoxic effect on both non-cancerous and cancerous cells with LC_50_ or IC_50_ greater than 533.33 μg/mL and 200 μg/mL, respectively. An exception was observed with the water leaf extract of *C. sylvaticus* that had good cytotoxicity (IC_50_ of 45.62 μg/mL) on MCF-7 cells with a promising selectivity index greater than 21.92 (see Table 3). On the other hand, ethanol leaf extracts of *C. pseudopulchellus* and *C. sylvaticus* were more cytotoxic on both non-cancerous and cancerous cells with lowest LC_50_ or IC_50_ values obtained against all cell lines. Acetone and ethanol leaf extracts of *C. pseudopulchellus* and *C. sylvaticus* had the highest cytotoxic activity on the non-cancerous cells with LC_50_ varying between 7.86 and 48.19 μg/mL while the acetone and ethanol extracts of *C. gratissimus* were less cytotoxic on these cell lines with LC_50_ varying between 152.30 and 462.88 μg/mL. The selectivity index (SI) values indicated that the acetone and ethanol extracts of *C. gratissimus* were most selective with SI ranging between 1.91 and 6.25 (see Table 3). In addition, the ethanol leaf extract and acetone leaf extract of *C. sylvaticus* were highly selective against A549 and MCF-7 cells with SI of 4.70 and 2.12, respectively. The same observation was made with the acetone leaf extract of *C. pseudopulchellus* which had SI of 1.31 and 1.95 against A549 and MCF-7 cells, respectively. On the contrary, the ethanol leaf extract of *C. pseudopulchellus* was less selective on non-cancerous cells with the lowest SI values ranging between 0.12 and 0.58 against all cancerous cells. Similarly, acetone and ethanol leaf extracts of *C. sylvaticus* were less selective with SI varying between 0.07 and 0.18 against Caco-2 and HeLa cells. Doxorubicin hydrochloride, the positive control, was highly cytotoxic on all cells with SI ranging between 0.87 and 1.75.

**Table 3:** Cytotoxic effect (IC_50_ and LC_50_) and the selectivity index (SI) of different extracts from *Croton* species and reference drug (doxorubicin hydrochloride) on cancerous cell lines versus a non-cancerous cell line

Plant Name	Extracts	LC_50_ (μg/mL)	IC_50_ (μg/mL) and Selectivity index = LC_50_/IC_50_							
		Vero	A549	SI	Caco-2	SI	HeLa	SI	MCF-7	SI
*Croton gratissimus*	CGA	462.88 ± 7.71^a^	97.46 ± 2.20^a^	4.75	74.05 ± 5.79^a^	6.25	78.21 ± 0.17^a^	5.91	83.74 ± 2.06^a^	5.52
	CGE	152.30 ± 3.68^b^	79.60 ± 2.32^b^	1.91	48.46 ± 3.47^b^	3.14	73.78 ± 4.12^a^	2.06	39.75 ± 2.49^b^	3.83
	CGW	533.33 ± 13.21^a^	> 200	<2.66	> 200	<2.66	> 200	<2.66	> 200	<2.66
*Croton pseudopulchellus*	CPA	48.19 ± 5.27^c^	36.54 ± 1.81^c^	1.31	112.74 ± 4.26^c^	0.42	128.69 ± 21.97^b^	0.37	24.65 ± 2.37^c^	1.95
	CPE	7.86 ± 1.47^d^	23.78 ± 1.41^d^	0.33	36.24 ± 2.34^d^	0.21	63.79 ± 1.02^c^	0.12	13.54 ± 1.18^d^	0.58
	CPW	> 1000	> 200	ND	> 200	ND	> 200	ND	> 200	ND
*Croton sylvaticus*	CSA	27.92 ± 0.62^e^	32.78 ± 2.55^c^	0.85	150.63 ± 8.79^e^	0.18	169.09 ± 13.05^b^	0.16	13.13 ± 2.76^d^	2.12
	CSE	8.23 ± 0.44^d^	1.75 ± 0.62^e^	4.70	103.73 ± 1.47^c^	0.08	106.52 ± 4.50^b^	0.07	6.02 ± 1.60^e^	1.36
	CSW	> 1000	> 200	ND	> 200	ND	> 200	ND	45.62 ± 5.69^b^	>21.92
Doxorubicin (μM)		1.90 ± 0.15^f^	1.30 ± 0.06^f^	1.46	1.08 ± 0.18^f^	1.75	2.17 ± 0.08^d^	0.87	1.11 ± 0.03^f^	1.71

Data are presented as means of triplicate measurements ± standard error; superscript letters a-f represent statistical difference between data obtained, and for each cell line within a column of the above table, data with different letters mean significantly different at p < 0.05 while data with same letters are statistically not different. ND = Not Determined. IC_50_: concentration required to inhibit the cell growth by 50% compared to untreated controls. SI is the selectivity index which is determined for each extract by dividing the LC_50_ on the non-cancerous cell by the IC_50_ on each cancer cell in the same units. CGA, CGE and CGW represent respectively acetone, ethanol and water extracts of *Croton gratissimus*. CPA, CPE and CPW represent respectively acetone, ethanol and water extracts of *Croton pseudopulchellus.* CSA, CSE and CSW represent respectively acetone, ethanol and water extracts of *Croton sylvaticus*

### Induction of caspase-dependent apoptosis by active extracts on cancerous cells

**Fig. 2:**
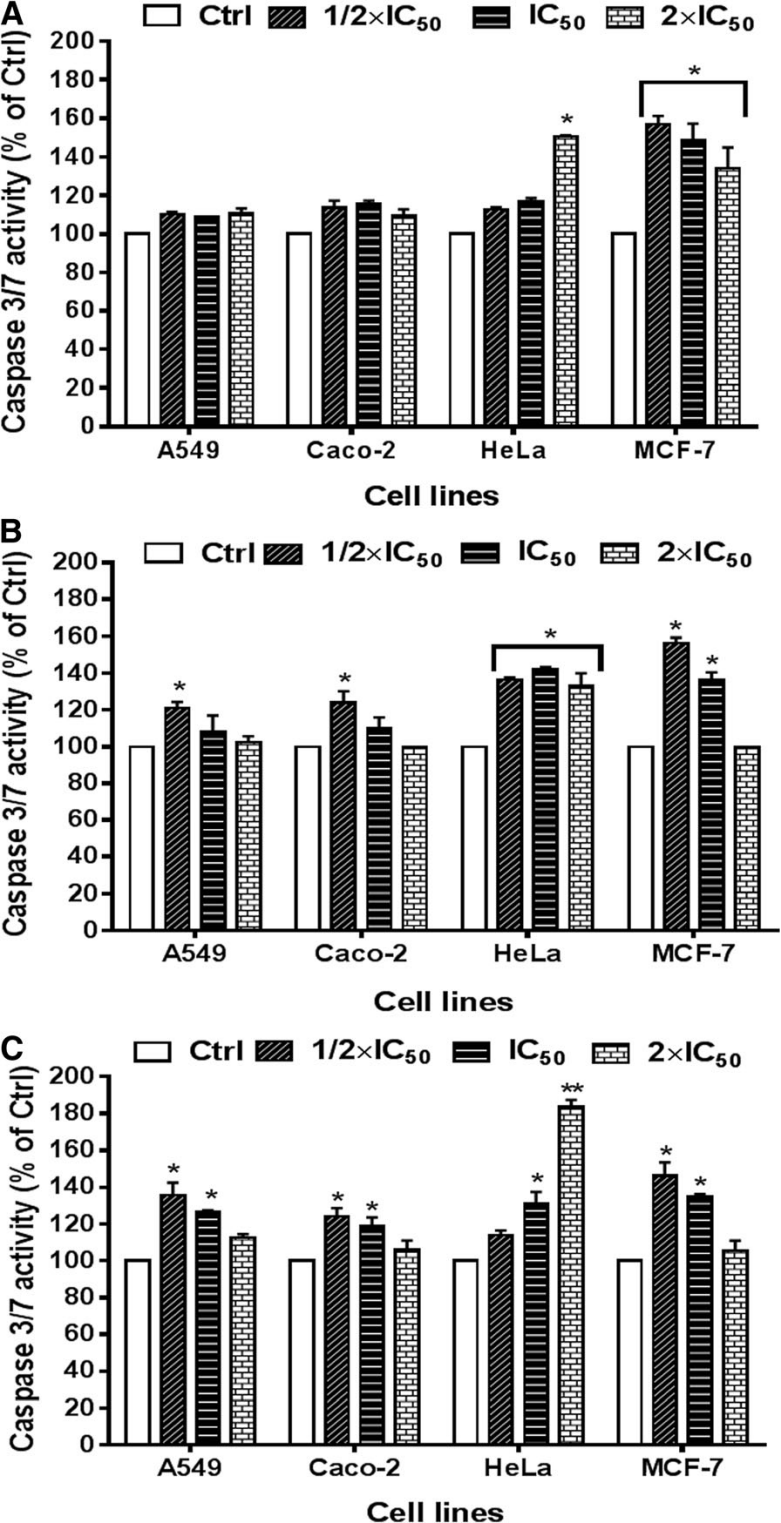
Activation of caspase-3/− 7 after 24 h of treatment with acetone leaf extracts of *Croton sylvaticus* (**a**), *Croton pseudopulchellus* (**b**) and *Croton gratissimus* (**c**) on cancerous A549, Caco-2, HeLa and MCF-7 cells. The caspase-3/− 7 activity is expressed as percentage or fold change to the untreated cells (control). Data are presented as mean ± standard error of three independent experiments. *P < 0.05 and **P < 0.01 indicate the significant difference compared to the control. CSA, CPA and CGA represent respectively acetone leaf extracts of *Croton sylvaticus*, *Croton pseudopulchellus* and *Croton gratissimus.* Ctrl: control group (0.5% DMSO)

In this assay, acetone leaf extracts of the three *Croton* species were used based on their high selectivity indexes or lower cytotoxicity to non-cancerous cells compared to other extracts. The activation of caspase-3 and -7 enzymes was differentially observed in all the four cancerous cells treated with the active extracts compared to the untreated controls (see Fig. 2). Caspase − 3 and − 7 enzymes were better activated after treatment with acetone leaf extracts of the three plants on HeLa and MCF-7 cells. The activation of these enzymes was also observed on A549 and Caco-2 cells only after treatment with the acetone leaf extracts of *C. pseudopulchellus* and *C. gratissimus* (Fig. 2b and c). These two extracts significantly (*P* < 0.05) induced caspase − 3 and − 7 activity in all cancerous cells at concentrations of ½ x IC_50_ (1.24 to 1.56-fold change). A non-significant increase of the activity of caspase − 3 and − 7 was noted after treatment with acetone leaf extracts of *C. sylvaticus* on A549 and MCF-7 cells (1.10 to 1.13-fold change). The acetone leaf extract of *C. gratissimus* induced activation of caspase − 3 and − 7 activity in a concentration-dependent manner on HeLa cells (Fig. 2c), and the highest induction (1.83-fold change) was obtained at the concentration of 2 x IC_50_.

## Discussion

Our study aimed to evaluate the antioxidant, anti-inflammatory and cytotoxic activities of three *Croton* species. The ethanol leaf extracts of the three plants were highly active in all experiments (except the NO inhibitory activity) compared to acetone and water leaf extracts. These results suggested that the antioxidant, anti-inflammatory and cytotoxic compounds extracted from the three plants are more concentrated in the ethanol leaf extract than in the acetone or water leaf extracts. We also investigated the potential relationship between the antioxidant, anti-inflammatory and cytotoxic activities of the active ethanol and acetone extracts. This relationship was analysed by determining the Pearson correlation coefficients (r) after plotting a linear curve with IC_50_ values of each cancer cell on the y-axis against phytochemical content or IC_50_ values of the antioxidant power (DPPH, ABTS) and anti-inflammatory activity (NO, 15-LOX) on the x-axis (Table 4). A linear correlation (r<0.5) existed between antioxidant, anti-inflammatory and cytotoxic activities, although this correlation was considered to be less strong. In fact, free radicals are well known to play a major role in the development of oxidative stress that can lead to many illnesses including cardiovascular diseases, diabetes, inflammation, degenerative diseases, and cancer [26]. Nitric oxide (NO), a molecule playing a crucial role in inflammatory response, can react with free radicals such as superoxides to produce peroxynitrites that can cause irreversible damage to cell membranes leading to the promotion of tumor growth and proliferation [27]. In addition, natural inhibitors of lipoxygenases have been shown to suppress carcinogenesis and tumor growth in a number of experimental models [28]. Moreover, several scientific reports have suggested that antioxidant and anti-inflammatory agents could be beneficial in the prevention and treatment of cancer [29]. Our results therefore suggest that the antioxidant or anti-inflammatory activities of extracts may contribute moderately to their cytotoxic activity. Phenolics and flavonoids are known for their contribution either directly or indirectly to the cytotoxic activity. In our study, we noted that the acetone and ethanol extracts of *C. gratissimus* which had the highest total phenolic contents (222.29 and 180.61 mgGAE/g respectively) were selectively cytotoxic to cancerous cells compared to non-cancerous. Indeed, due to their anti- and pro-oxidant potential, phenolics (which also include flavonoids) may have cytotoxic activity against different human cancer cells with little or no effect on normal cells. This selectivity in the cytotoxicity properties of phenolics has strengthened interest in formulating novel and less toxic anticancer products based on these types of compounds [30, 31].

**Table 4:** Correlation between phytochemical content, antioxidant, anti-inflammatory and antiproliferative activity of active extracts

Cell lines	Pearson correlation coefficient (r)					
	TPC	TFC	DPPH	ABTS	NO	15-LOX
A549	0.3530	0.2404	0.2850	0.4030	0.2525	0.1935
Caco-2	0.0143	0.4529	0.4545	0.2758	0.2820	0.2798
HeLa	0.0920	0.3724	0.3582	0.2043	0.2847	0.2251
MCF-7	0.3111	0.2399	0.0800	0.0003	0.2027	0.2341

Correlation coefficients were determined by plotting a linear curve with IC_50_ values of extracts obtained for each cancer cell on the y-axis against the corresponding phytochemical content or IC_50_ values of the antioxidant power (DPPH, ABTS) and anti-inflammatory activity (NO, 15-LOX) on the x-axis. NO: nitric oxide, 15-LOX: 15-lipoxygenase. TPC: total phenolic content, TFC: total flavonoid content, DPPH: 2,2-diphenyl-1-picrylhydrazyl radical, ABTS: 2,2′-azino-bis (3-ethylbenzothiazoline-6-sulfonic acid) radical

The goal of any chemotherapeutic treatment is to selectively attenuate or destroy pathogenic micro-organisms or cancerous cells with minimal side effects to the host cells [32]. This principle, known as selective toxicity, is the key to all chemotherapeutic treatment. In this study, the acetone and ethanol extracts of *C. gratissimus* were more selective with SI ranging between 1.91 and 6.25, and it therefore indicates that these extracts may be useful in the search for anticancer compounds. A cembranolide isolated from stem bark of *Croton gratissimus* had moderate activity against PEO1 and PEO1TaxR ovarian cancer cell lines [16]. In the present work, four cancerous (A549, Caco-2, HeLa, MCF-7) cells and a non-cancerous (Vero) cell line were used to evaluate the antiproliferative activity of the crude extracts from three *Croton* species. The use of these cancerous cells with the non-cancerous (Vero) cell line as cell models has been reported for comparison and determination of the selectivity indexes [33, 34]. However, the cytotoxic effect on this non-cancerous (Vero) cell line of animal origin needs to be confirmed on other non-cancerous cells of human origin. The selective toxicity of acetone and ethanol extracts of *C. gratissimus* also suggested that the active compounds interact with special cancer-associated receptors or cancer cell special molecule (not found in non-cancerous cells), thus activating some mechanisms that cause cancer cell death [35]. The activation of caspase − 3 and − 7 enzymes was observed in all four of the cancer cell types treated with the active extracts compared to the untreated cells, which therefore reveals that apoptosis has taken place in the treated cells. Indeed, caspases − 3, and − 7 are known as “executioners” of apoptosis since they serve as substrates for initiator caspases in extrinsic or intrinsic apoptotic pathways [36]. It will be important to comprehensively investigate the mechanism of the activity, and this aspect will be addressed once the compounds responsible for the activity have been isolated. The aim of the current study was to explore the possibility that extracts have inhibitory activity on cancer cell growth.

According to the United States National Cancer Institute, a crude extract is generally considered to have in vitro cytotoxic activity if the IC_50_ is lower than 30 μg/mL [37]. Based on this statement, acetone and ethanol extracts of *C. pseudopulchellus* and *C. sylvaticus* were considered as more active on both cancerous A549 and MCF-7 cells. Differences in the selectivity indexes of these extracts on these two cancerous cells may be ameliorated through the isolation of active compounds which might reduce the toxic effects of the crude extracts. Studies are ongoing to isolate active compounds from these active extracts.

## Conclusion

In summary, due to their selective toxicity between non-cancerous and cancerous cells, with beneficial antioxidant and anti-inflammatory activities, the acetone and ethanol leaf extracts of *Croton gratissimus* may be useful against cancer and other oxidative stress related diseases. The isolation of active compounds from this extract will be of great interest to fully understand the mechanism of anticancer activity. In addition, acetone and ethanol extracts of *C. pseudopulchellus* and *C. sylvaticus*, which were cytotoxic to both cancerous and non-cancerous cells, may be further explored as sources of new cytotoxic compounds.

## Additional files

Additional file 1: **Figure S1.** Non-linear regression curves for IC_50_ determination of different extracts from *Croton* species in 15-lipoxygenase (15-LOX) inhibitory assay. CSA and CSE represent respectively acetone, ethanol and water extracts of *Croton sylvaticus.* CGA and CGE represent respectively acetone, ethanol and water extracts of *Croton gratissimus.* CPA and CPE represent respectively acetone, ethanol and water extracts of *Croton pseudopulchellus.* (TIF 109 kb)

Additional file 2: **Figure S2.** Concentration-dependent graph of A549 cell viability of different extracts from *Croton* species**.** Extracts were tested at concentrations between 200 and 6.25 μg/mL; Ctrl: 0.5% DMSO. (TIF 128 kb)

Additional file 3: **Figure S3.** Concentration-dependent graph of Caco-2 cell viability of different extracts from *Croton* species**.** Extracts were tested at concentrations between 200 and 6.25 μg/mL; Ctrl: 0.5% DMSO. (TIF 156 kb)

Additional file 4: **Figure S4.** Concentration-dependent graph of HeLa cell viability of different extracts from *Croton* species. Extracts were tested at concentrations between 200 and 6.25 μg/mL; Ctrl: 0.5% DMSO. (TIF 142 kb)

Additional file 5: **Figure S5.** Concentration-dependent graph of MCF-7 cell viability of different extracts from *Croton* species**.** Extracts were tested at concentrations between 200 and 6.25 μg/mL; Ctrl: 0.5% DMSO. (TIF 136 kb)

Additional file 6: **Figure S6.** Concentration-dependent graph of Vero cell viability of different extracts from *Croton* species. Extracts were tested at concentrations between 1000 and 50 μg/mL Ctrl: 0.5% DMSO. (TIF 132 kb)

## Abbreviations

ABTS: 2,2′-azino-bis (3-ethylbenzothiazoline-6-sulfonic acid
ATCC: American type culture collection
DMSO: Dimethyl sulphoxide
DPPH: 2,2-diphenyl-1-picrylhydrazyl
FOX: Ferrous oxidation-xylenol orange
GAE: Gallic acid equivalent
IC_50_: Inhibitory concentration to 50% of cells
LC_50_: Lethal concentration to 50% of cells
LOX: Lipoxygenase
LPS: Lipopolysaccharide
MEM: Minimal essential medium
MTT: 3-(4,5-dimethylthiazol-2-yl)-2,5-diphenyltetrazolium bromide
NO: Nitric oxide
QE: Quercetin equivalent
TFC: Total flavonoid content
TPC: Total phenolic content
