# Supplementary figures and images for: Croton gratissimus leaf extracts inhibit cancer cell growth by inducing caspase 3/7 activation with additional anti-inflammatory and antioxidant activities
Source: BMC Complement Altern Med. 2018 Nov 14;18:305. doi: 10.1186/s12906-018-2372-9 (PMC6236960; doi:10.1186/s12906-018-2372-9)

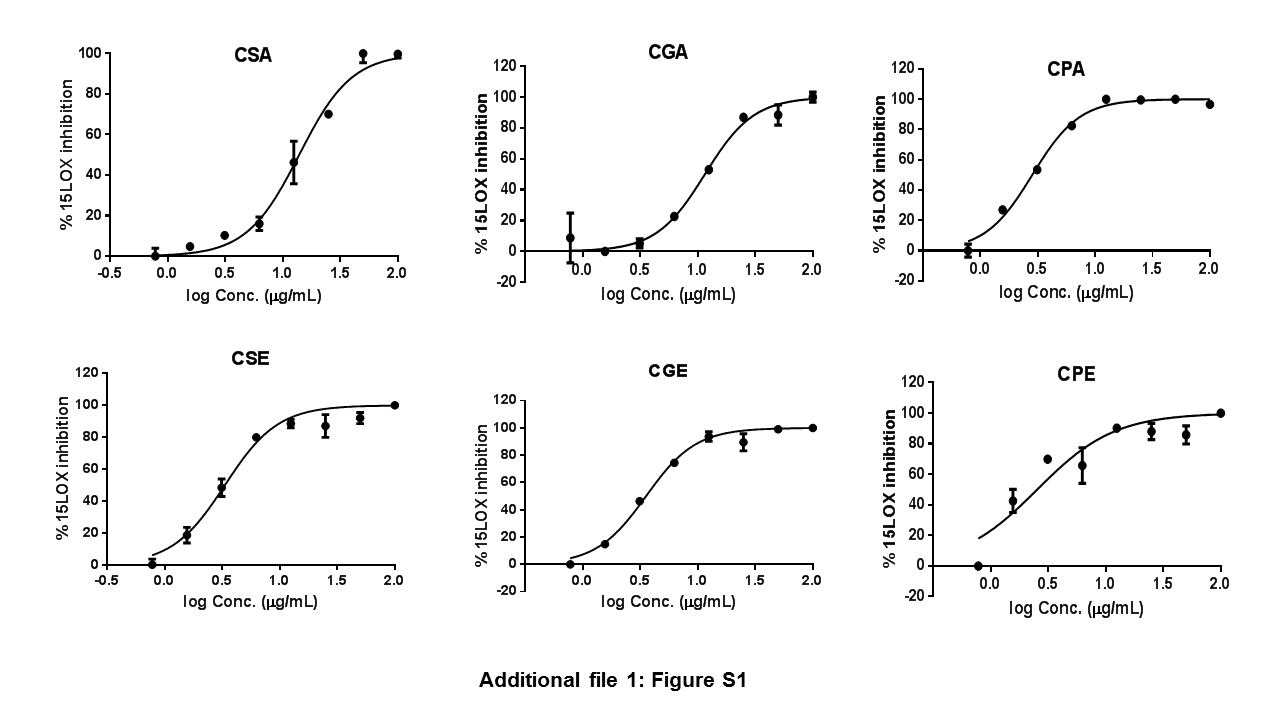

Supplement: Supplementary file 1 — Figure S1. Non-linear regression curves for IC50 determination of different extracts from Croton species in 15-lipoxygenase (15-LOX) inhibitory assay. CSA and CSE represent respectively acetone, ethanol and water extracts of Croton sylvaticus. CGA and CGE represent respectively acetone, ethanol and water extracts of Croton gratissimus. CPA and CPE represent respectively acetone, ethanol and water extracts of Croton pseudopulchellus. (TIF 109 kb) [file 12906_2018_2372_MOESM1_ESM.tif]

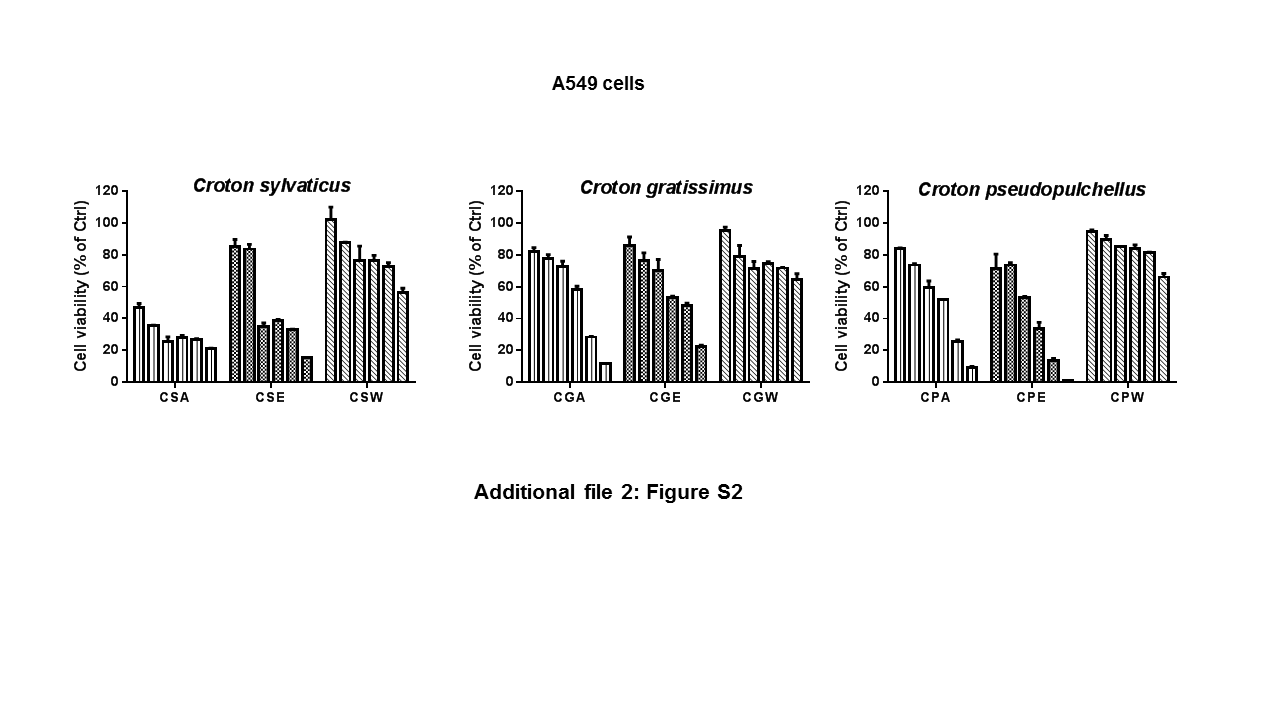

Supplement: Supplementary file 2 — Figure S2. Concentration-dependent graph of A549 cell viability of different extracts from Croton species. Extracts were tested at concentrations between 200 and 6.25 μg/mL; Ctrl: 0.5% DMSO. (TIF 128 kb) [file 12906_2018_2372_MOESM2_ESM.tif]

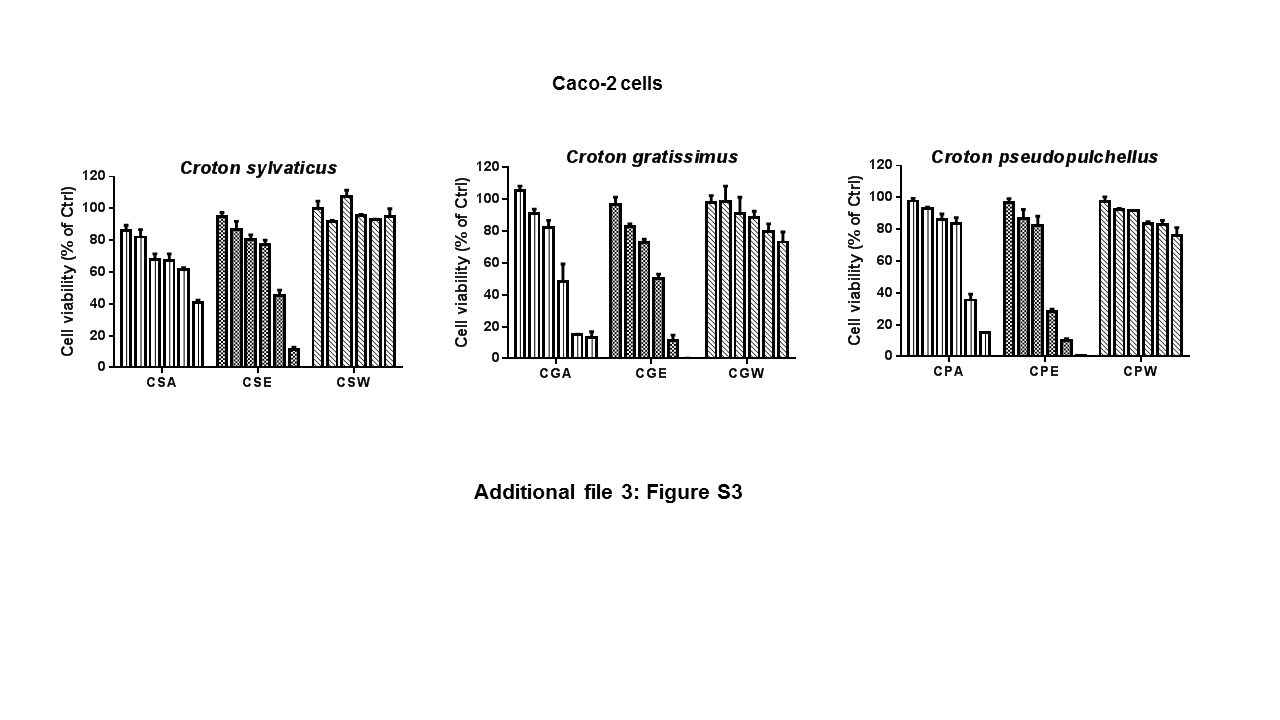

Supplement: Supplementary file 3 — Figure S3. Concentration-dependent graph of Caco-2 cell viability of different extracts from Croton species. Extracts were tested at concentrations between 200 and 6.25 μg/mL; Ctrl: 0.5% DMSO. (TIF 156 kb) [file 12906_2018_2372_MOESM3_ESM.tif]

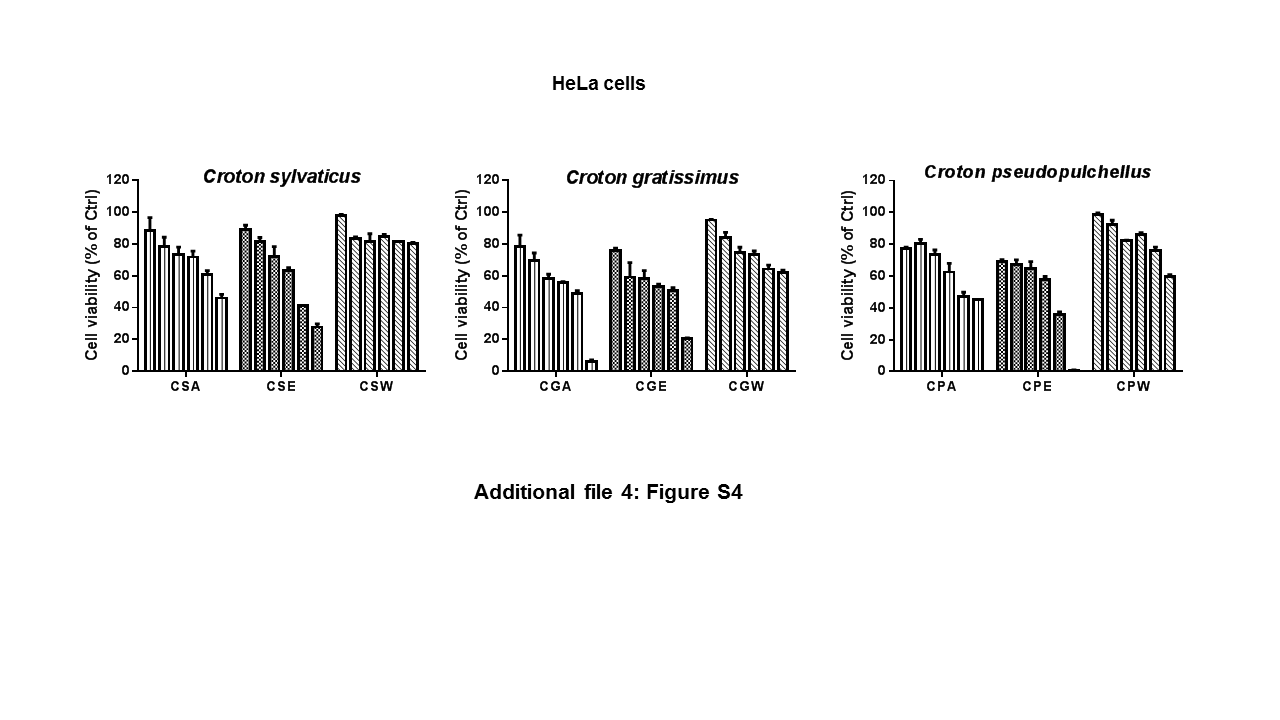

Supplement: Supplementary file 4 — Figure S4. Concentration-dependent graph of HeLa cell viability of different extracts from Croton species. Extracts were tested at concentrations between 200 and 6.25 μg/mL; Ctrl: 0.5% DMSO. (TIF 142 kb) [file 12906_2018_2372_MOESM4_ESM.tif]

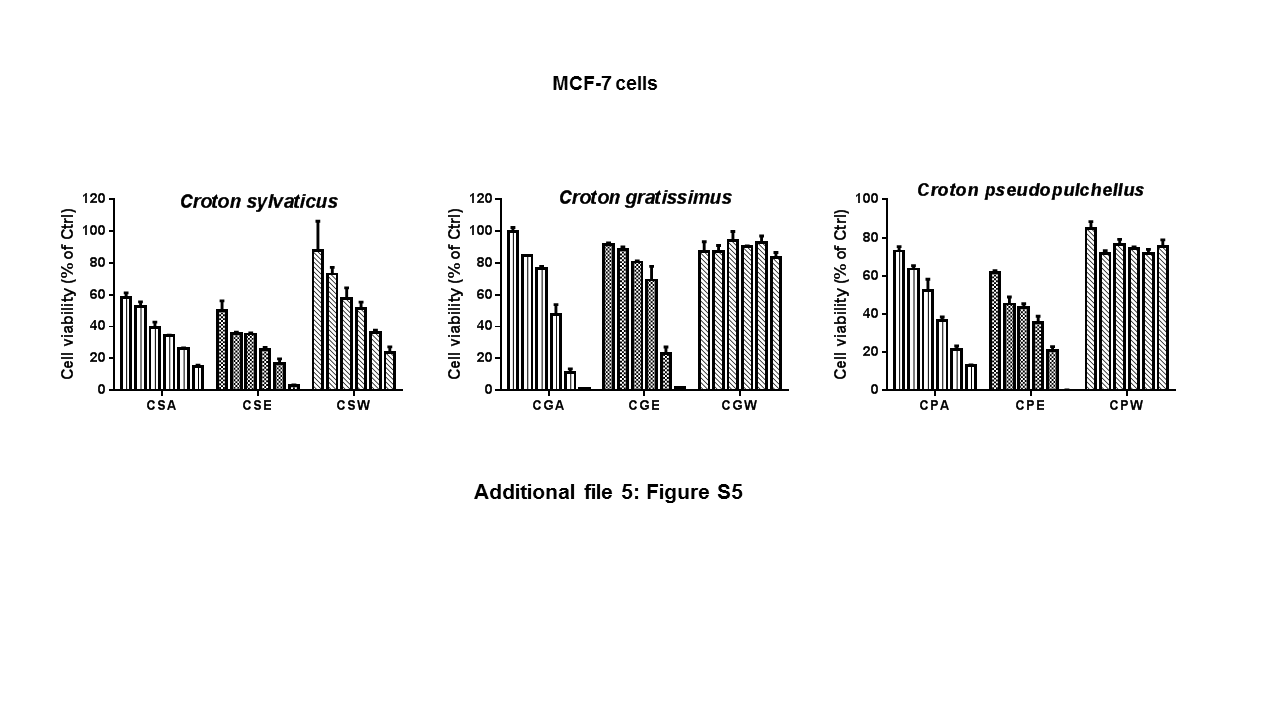

Supplement: Supplementary file 5 — Figure S5. Concentration-dependent graph of MCF-7 cell viability of different extracts from Croton species. Extracts were tested at concentrations between 200 and 6.25 μg/mL; Ctrl: 0.5% DMSO. (TIF 136 kb) [file 12906_2018_2372_MOESM5_ESM.tif]

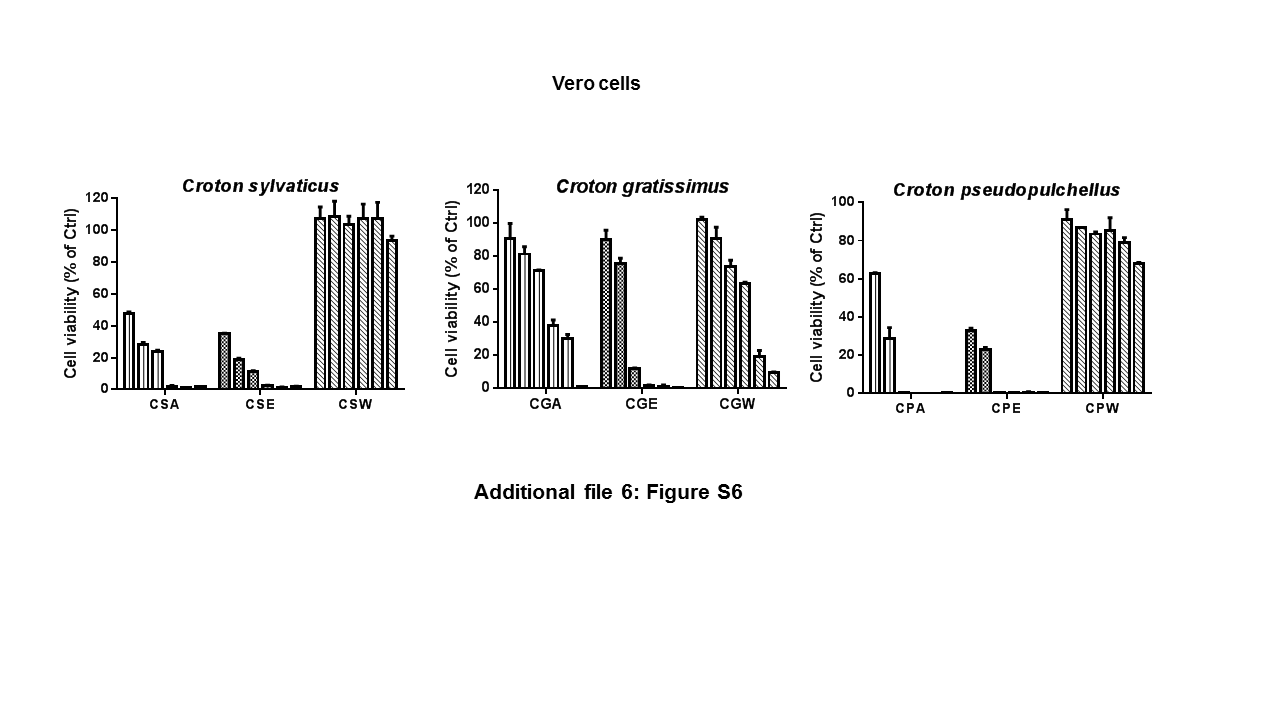

Supplement: Supplementary file 6 — Figure S6. Concentration-dependent graph of Vero cell viability of different extracts from Croton species. Extracts were tested at concentrations between 1000 and 50 μg/mL Ctrl: 0.5% DMSO. (TIF 132 kb) [file 12906_2018_2372_MOESM6_ESM.tif]
